# A Novel Nonsense Mutation of the AGL Gene in a Romanian Patient with Glycogen Storage Disease Type IIIa

**DOI:** 10.1155/2016/8154910

**Published:** 2016-01-17

**Authors:** Anca Zimmermann, Heidi Rossmann, Simona Bucerzan, Paula Grigorescu-Sido

**Affiliations:** ^1^Department of Endocrinology and Metabolic Diseases, 1st Clinic of Internal Medicine, University of Mainz, Langenbeckstrasse 1, 55131 Mainz, Germany; ^2^Institute for Clinical Chemistry and Laboratory Medicine, University of Mainz, Langenbeckstrasse 1, 55131 Mainz, Germany; ^3^Center of Genetic Diseases, Emergency Children's Hospital, University of Medicine and Pharmacy, Motilor Street 68, 400370 Cluj, Romania

## Abstract

*Background*. Glycogen storage disease type III (GSDIII) is a rare metabolic disorder with autosomal recessive inheritance, caused by deficiency of the glycogen debranching enzyme. There is a high phenotypic variability due to different mutations in the* AGL* gene.* Methods and Results*. We describe a 2.3-year-old boy from a nonconsanguineous Romanian family, who presented with severe hepatomegaly with fibrosis, mild muscle weakness, cardiomyopathy, ketotic fasting hypoglycemia, increased transaminases, creatine phosphokinase, and combined hyperlipoproteinemia. GSD type IIIa was suspected. Accordingly, genomic DNA of the index patient was analyzed by next generation sequencing of the AGL gene. For confirmation of the two mutations found, genetic analysis of the parents and grandparents was also performed. The patient was compound heterozygous for the novel mutation c.3235C>T, p.Gln1079^⁎^ (exon 24) and the known mutation c.1589C>G, p.Ser530^⁎^ (exon 12). c.3235 >T, p.Gln1079^⁎^ was inherited from the father, who inherited it from his mother. c.1589C>G, p.Ser530^⁎^ was inherited from the mother, who inherited it from her father.* Conclusion*. We report the first genetically confirmed case of a Romanian patient with GSDIIIa. We detected a compound heterozygous genotype with a novel mutation, in the context of a severe hepatopathy and an early onset of cardiomyopathy.

## 1. Introduction

Glycogen storage disease type III (GSDIII), sometimes referred to as Cori-Forbes disease (OMIM 232400), is a metabolic disorder with autosomal recessive inheritance, caused by glycogen debranching enzyme (GDE) deficiency, with accumulation of an intermediate glycogen form called limit-dextrin (LD) in affected tissues [1].

GDE contains two catalytic sites with two different functions: 4-alpha-glucanotransferase (EC 2.4.1.25) and amylo-1,6-glucosidase (EC 3.2.1.33) [2–4].

*AGL* (amylo-alpha-1, 6-glucosidase, 4-alpha-glucanotransferase), the gene encoding GDE, spans 85 kb of genomic DNA, contains 35 exons [3], and is located on chromosome 1p21.2 [5]. Bao et al. recognized the presence of six different isoforms of GDE that differ in the 5′ end [6]. Transcript variant 1 of the* AGL* gene (NM_000642.2), which is mainly expressed in liver and kidney, consists of 34 exons, 33 of which are coding. Tissue-specific alternative splicing may contribute to the wide range of enzymatic and clinical variability described for GSDIII mutations (Human Gene AGL (uc001dsi.1); https://genome.ucsc.edu/). The glycogen binding site is encoded by exons 31 and 32 and the active site is encoded by exons 6, 13, 14, and 15 [7].

GDE deficiency leads to storage of LD in affected tissues (liver, skeletal muscles, and myocardium), with morphological and functional consequences. There are four subtypes of GSDIII, depending on the type of enzymatic deficiency and its location. The most frequent two subtypes are caused by the deficiency of both catalytical GDE functions, with involvement of liver and muscle in GSDIIIa (85% of patients) or only of liver in GSDIIIb (15% of patients) [8].

The clinical picture varies according to age. In infants, hepatomegaly, keto-hypoglycemic episodes, muscular hypotonia, and growth retardation occur, accompanied by highly increased transaminases, combined hyperlipoproteinemia, and increased values of serum creatine kinase. In adults with the subtype IIIa the main findings are progressive myopathy, cardiomyopathy, and sometimes altered hepatic tests [9, 10].

The number of known mutations associated with GSDIII has increased over time, with 130 mutations by the end of 2014 [11, 12].

We report on a Romanian child with a GSDIIIa phenotype, harbouring a new nonsense mutation (c.3235C>T; p.Gln1079^*∗*^) in a compound heterozygote state with a previously known mutation (c.1589C>G; pSer530^*∗*^). For correct segregation, molecular analysis has also been performed on the parents and the two pairs of grandparents.

## 2. Case Presentation

A 2.3-year-old boy was admitted to the Department of Genetic Diseases of the Emergency Hospital for Children in Cluj, Romania, for evaluation of hepatomegaly and elevated transaminases. The patient was the parents' first child, born from the second gestation, after an initial spontaneous abortion in the 8th gestational week, from apparently healthy, nonconsanguineous, and young parents (age at the child's birth: mother 24 yrs, father 27 yrs). The pregnancy was normal, with spontaneous vaginal delivery at term. The newborn appeared healthy, with a length of 56 cm (97. percentile) and weight of 3500 g (50. percentile) [13]. At the age of 1 year, hepatomegaly and highly increased transaminases were observed. A metabolic storage disorder was suspected and the patient was referred to our clinic for further investigation.

At admission to our service, the patient was in good general condition. He presented with a body height of 90.0 cm (50. percentile) and a body weight of 16.5 kg (97. percentile) [13], severe hepatomegaly, mild muscle weakness, and mild splenomegaly. On sonographic volumetric evaluation, the hepatomegaly was 4.3x the upper normal limit (UNL) and the splenomegaly was 1.3x UNL. Normal values were considered to be 2.5% of the patient's weight for the liver and 0.2% of the patient's weight for the spleen, according to published criteria [14]. Hepatic sonography showed, additionally, a slightly increased echogenic pattern.

Laboratory tests showed the following abnormalities: increased transaminases (alanine transaminase (ALT) = 760 UI/L and aspartate transaminase (AST) = 767 UI/L; normal values 20–40 UI/L) and gamma-glutamyl transferase (*γ*GT) = 333 UI/L; normal values < 20 UI/L; viral markers for hepatitis B and hepatitis C were negative; moderately increased creatine phosphokinase (CPK) = 545 UI/L (normal values 30–200 UI/L) and lactic dehydrogenase (LDH) = 780 UI/L (normal values 120–300 UI/L), confirming muscular involvement in accordance with the clinical picture; ketotic fasting hypoglycemia (48 mg/dL) with metabolic acidosis (pH = 7.26, BE = −12.6, HCO_3_^−^ = 12.8 mmol/L); and combined hyperlipoproteinemia, with total cholesterol = 282 mg/dL (normal values for age ≤ 170 mg/dL), triglycerides = 300 mg/dL (normal values for age ≤ 100 mg/dL).

Liver biopsy was performed before the patient's referral to our clinic. The biopsy showed enlarged hepatocytes with intracytoplasmic glycogen loading and stellate as well as periportal bridging fibrosis with incomplete nodular transformation. Cardiac ultrasound showed a hypertrophic obstructive myocardiopathy with biventricular hypertrophy on electrocardiogram (EKG).

The clinical picture and the diagnostic findings suggest a hepatic glycogenosis (type IIIa).

### 2.1. Genetic Testing

All of the genetic investigations performed on this patient and his family members were done after informed consent was obtained following local Institutional Review Board policies and procedures.

The genomic DNA of the index patient, isolated from peripheral blood leucocytes, was analyzed by a next generation sequencing panel (Centogene AG, Rostock, Germany), comprising the entire coding region and the highly conserved exon-intron splice junctions of the* AGL* gene (amylo-alpha-1,6-glucosidase, 4-alpha-glucanotransferase, RefSeq NM_000642.2 [variant 1], NM_000645.2 [variant 5], NM_000646.2 [variant 6]);* G6PC* (glucose-6-phosphatase, catalytic subunit, RefSeq NM_000151.3);* GBE1* (glucan (1,4-alpha), branching enzyme 1, NM_000158.3) and* SLC37A4* (solute carrier family 37 (glucose-6-phosphate transporter, member 4), RefSeq NM_001164278.1). Library preparation was based on polymerase chain reaction (PCR) amplicons and the minimal coverage was 30x.

**Figure 1:**
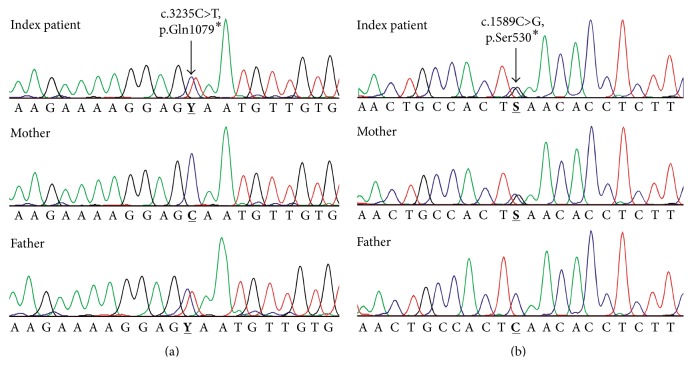
Sequence analysis of the index patient showing the novel mutation c.3235C>T, p.Gln1079^*∗*^ in exon 24 (a) and the known mutation c.1589C>G, p.Ser530^*∗*^ in exon 12 (b).

A previously unreported heterozygous variant in exon 24 of the* AGL* gene was identified: c.3235C>T (p.Gln1079^*∗*^); see Figure 1(a). This variant causes the reading frame to be interrupted by a premature stop codon and is classified, according to the American College of Medical Genetics and Genomics (ACMG) recommendations, as class 2, a sequence variation previously unreported and of the type that is expected to cause the disorder [15]. Furthermore, the mutation c.1589C>G (p.Ser^*∗*^) in exon 12 of the* AGL* gene has also been detected in a heterozygous state (Figure 1(b)). This mutation has been previously described as disease-causing.

No disease-causing mutation was detected in the* G6PC*,* GBE1*, or* SLC37A4* genes.

The results were confirmed in a second independent sample at the Institute for Clinical Chemistry and Laboratory Medicine (Mainz, Germany) by conventional sequencing of exons 12 and 24 (PCR primers: ACCAGTGTTTCCTTGAAGTAATTG and AAATCAATGCTTGTGTCCAACTAG for amplification of exon 12 and TTGAAGGAAAGAAACCAAGTAAA and CTTGAGTAGCATTACAAGCTTTT for exon 24). For sequencing (Dye Terminator Cycle Sequencing Quick Start Kit, CEQ 8000 Genetic Analysis System; Beckman Coulter, Krefeld, Germany) M13/M13R tags were added to the PCR primers.

Given the autosomal recessive mode of inheritance of glycogen storage disease type III, we performed parental carrier testing to confirm the mutation phase (cis or trans). We tested the grandparents, too. The mutation analysis of family members has also been performed in the Institute for Clinical Chemistry and Laboratory Medicine (Mainz, Germany).

**Figure 2:**
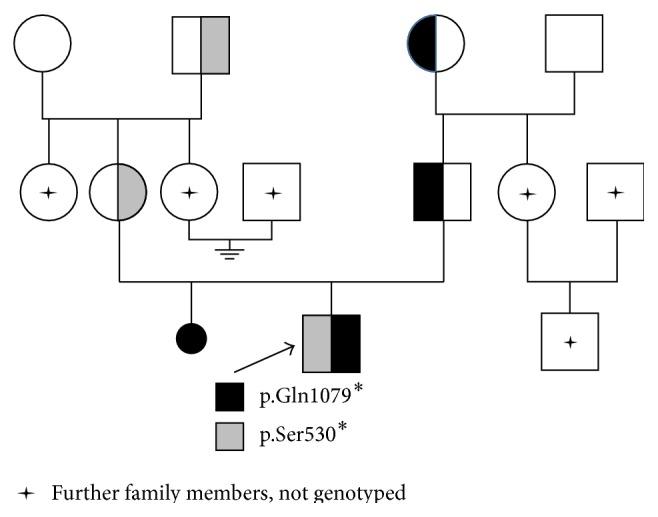
Diagnostic sequence analysis of the AGL gene (exons 12 and 24) shows compound heterozygosity for c.1589C>G, p.Ser530^*∗*^ and c.3235C>T, p.Gln1079^*∗*^ in the index patient, and heterozygosity for either c.1589C>G, p.Ser530^*∗*^ or c.3235C>T, p.Gln1079^*∗*^ in his parents and grandparents.

The results demonstrated the inheritance of the two mutations in trans phase. The novel mutation, c.3235C>T (p.Gln1079^*∗*^), was inherited from the father, who inherited it from his mother, while the known mutation c.1589C>G (p.Ser530^*∗*^) was inherited from the mother, who inherited it from her father (Figure 2). Therefore, the index patient has a compound heterozygous genotype with the novel mutation c.3235C>T (p.Gln1079^*∗*^) in exon 24 and the known mutation c.1589C>G (p.Ser530^*∗*^) in exon 12 (Figures 1(a) and 1(b)).

### 2.2. Follow-Up

The therapy was according to present recommendations with a specific diet including frequent meals, high in carbohydrates; supplementation of maltodextrin (1 g/kg/4 hrs) during the night to prevent hypoglycemia; protein enrichment during the day (up to 3 g/kg).

Six months after the start of treatment, we registered no more hypoglycemia, improvement of dyslipidemia (total cholesterol from 282 to 164 mg/dL and triglycerides from 300 to 184 mg/dL), and decrease of transaminases (ALT from 760 to 529 U/L; AST from 767 to 472 U/L) with constant values of gamma-GT.

## 3. Discussion

The majority of the mutations reported in the AGL gene to date are nonsense or missense mutations, small or large deletions, or insertions. Very few mutations are specific for a geographical region; most of them are private mutations [11, 12]. Three mutations (p.Arg864^*∗*^, p.Arg1228^*∗*^, and p.Trp680^*∗*^) account for approximately 28% of the known mutations in individuals of European origin [11].

Mutations with a certain regional pattern are homozygous in affected individuals and have been described in Inuit children from the Eastern region of the Hudson bay and in Jews of North-African origin (c.4555delT) [12, 16], in inhabitants of the Faroe islands (c.1222C>T; p.R408^*∗*^) [17] and in Tunisian patients: c.3216_3217delGA [18] and p.W1327^*∗*^ [19]. These findings are explained by a “founder effect” and are responsible for a high prevalence of the disease in these areas: 1/5,420 in North-African Jews, 1/3,600 in the inhabitants of the Faroe islands, and 1/2,500 in Inuits versus 1/100,000 in North-America [17, 18]. A higher prevalence with clustering of some mutations has been reported from Japan [20] and Korea [21].

The genetic heterogeneity due to a high number of private mutations precludes a diagnostic strategy based on screening for the most common ones. Furthermore, the heterogeneity of the clinical picture, with high phenotypic variability in patients with the same genotype, makes genotype-phenotype correlations extremely difficult [10, 11]. Heterogeneity even within a given family has been noted [9]. The only observed correlation is the association between mutations in exon 3 with GSD type IIIb [22].

The index patient reported here has a GSD type III phenotype, with a pronounced hepatic involvement (severe hepatomegaly, altered liver tests, and liver fibrosis) and a hypertrophic obstructive cardiomyopathy. The association of a mild muscular involvement suggests GSD type IIIa. The absence of a mutation in exon 3, associated with GSD type IIIb, supports the diagnosis. As shown, our patient displayed a compound heterozygous genotype c3235C>T (p.Gln1079^*∗*^)/c.1589C>G (p.Ser530^*∗*^). c.3235C>T (p.Gln1079^*∗*^) is a novel mutation leading to a stop codon, yielding a truncated protein lacking the 3′ 454 amino acids. The protein lacks exon 31, which is one glycogen binding area. The second mutation, c1589C>G, (p.Ser530^*∗*^), also leads to a premature stop codon and has been described previously in a patient of Mediterranean origin, who was compound heterozygous and presented with a severe phenotype [23].

Some studies describe severe hepatopathy at older ages in patients with GSD III, such as in the second decade of life in an 18-year-old patient with the genotype c.2607_2610delATCC/c.1672dupA [24] or in the third decade in 16% of the patients reported [9]. The cardiomyopathy is also reported later, in adult life [25]. The phenotype in our patient with early hepatic and cardiac damage may be explained by the fact that both mutations generate premature stop codons, with the theoretical risk of null alleles for the development of a more severe clinical picture [9]. It is possible that further improvement will occur in time, so the severity of the phenotype will be shown in future. Nevertheless, the severe histologic damage of the liver remains a key element of concern.

## 4. Conclusions

We report on a new nonsense mutation (c.3235C>T; p.Gln.1079^*∗*^ in exon 24 of the* AGL* gene), in a compound heterozygote state with the known mutation c1589C>G (p.Ser530^*∗*^) in the first genetically confirmed Romanian patient with GSDIIIa. Our observation adds to many other previous reports, pointing out to the heterogenous genetic background of the disease and the need for complete* AGL* gene sequencing in the setting of a suggestive clinical picture, in order to confirm the diagnosis, to screen further siblings and to offer correct genetic counselling in young families.
